# Tumor‐Induced Splenic Remodeling: Mechanisms of Systemic Immunosuppression and Emerging Therapeutic Opportunities

**DOI:** 10.1002/advs.202600027

**Published:** 2026-03-20

**Authors:** Yuehua Liu, Xiaoqian Nie, Xiaofei Gao

**Affiliations:** ^1^ Research Center for Industries of the Future and School of Life Sciences Westlake University Hangzhou Zhejiang China; ^2^ Westlake Laboratory of Life Sciences and Biomedicine Hangzhou Zhejiang China; ^3^ Key Laboratory of Growth Regulation and Translational Research of Zhejiang Province, School of Life Sciences Westlake University Hangzhou Zhejiang China

**Keywords:** immunosuppression, myeloid‐derived suppressor cells, spleen‐targeted therapy, tumor‐induced splenic remodeling

## Abstract

Despite the transformative impact of cancer immunotherapies such as immune checkpoint blockade, durable clinical responses remain limited. Increasing evidence indicates that antitumor immunity is governed not only by the tumor microenvironment, but also by systemic immune regulation mediated by peripheral immune organs. Among these, the spleen functions as a central blood‐filtering immune hub that integrates immune activation with hematopoietic adaptation. During tumor progression, the spleen undergoes profound remodeling, shifting from a site of immune surveillance to a pro‐tumorigenic immune compartment characterized by pathological extramedullary hematopoiesis, and sustained generation of immunosuppressive cell populations, including myeloid‐derived suppressor cells. Continuous systemic export of these cells reinforces tumor immune evasion and constrains the efficacy of immune checkpoint blockade. In this review, we summarize current understanding of splenic structure and immunological function, delineate the mechanisms driving tumor‐induced splenic remodeling, and examine its role in systemic immunosuppression and resistance to cancer immunotherapy. We further evaluate emerging therapeutic strategies aimed at targeting the spleen, highlighting both their translational potential and key biological barriers. Collectively, this work identifies tumor‐induced splenic remodeling as a central yet underappreciated determinant of immunotherapy response and a promising target for next‐generation immunotherapies.

## 1 Introduction

Cancer immunotherapy, particularly immune checkpoint blockade (ICB) therapy, has revolutionized the landscape of clinical oncology in recent years. By targeting immune checkpoint molecules such as PD‐1/PD‐L1 or CTLA‐4, ICB therapy can reinvigorate the anti‐tumor functions of T cells and has demonstrated durable clinical benefits across multiple malignancies [1, 2, 3]. Despite the clinical success of ICB, the majority of patients do not benefit from this therapy. Objective response rates to PD‐1/PD‐L1 inhibitors range from 20% to 30% across most solid tumors (primary resistance) [4, 5], and a substantial proportion of initial responders eventually experience disease relapse as treatment progresses (secondary resistance) [6]. The development of resistance is closely associated with the immunosuppressive characteristics of the tumor microenvironment (TME) [7]. Nevertheless, accumulating evidence indicates that dynamic regulation of the peripheral immune system, particularly the spleen, systemically shapes the intensity and quality of anti‐tumor immune responses by continuously supplying effector or regulatory cells to tumor tissues [8, 9].

The spleen plays a paradoxical dual role in shaping anti‐tumor immunity. On one hand, as a crucial secondary lymphoid organ, it serves as a site for the activation and differentiation of T cells, B cells, and NK cells, playing a central role in initiating and maintaining systemic anti‐tumor immunity [10]. Notably, dynamic changes in T cell clonal repertoires within the peripheral immune pool may determine the ultimate efficacy of ICB. Single‐cell sequencing and T cell receptor (TCR) tracking studies have revealed a “T cell clonal replacement model.” During ICB therapy, new anti‐tumor T cell clones are generated in the periphery. These cells progressively replace exhausted T cells in the TME and directly contribute to tumor control [11, 12, 13]. These findings provide strong support for the pivotal role of peripheral immune organs in cancer immunotherapy. On the other hand, the spleen also serves as a source of immunosuppressive cells, including myeloid‐derived suppressor cells (MDSCs) and regulatory T cells (Tregs). During tumor progression, it undergoes functional remodeling driven by tumor‐derived factors (TDFs) such as granulocyte‐macrophage colony‐stimulating factor (GM‐CSF) [14], vascular endothelial growth factor (VEGF) [15], and Interleukin‐6 (IL‐6) [16], leading to extramedullary hematopoiesis (EMH) and expansion of MDSCs, which actively suppress anti‐tumor immunity and impair the efficacy of ICB [17]. However, despite the increasing recognition of the importance of peripheral organs such as the spleen in anti‐tumor immunity, targeted interventions for these organs are still in the early stages of exploration [18, 19]. This limitation is largely constrained by the biological barrier properties of peripheral immune organs and the inability of current delivery systems to efficiently and specifically target these sites [19, 20].

In this review, we propose that tumor‐induced splenic remodeling represents a systemic immunosuppressive mechanism that works together with the TME to influence anti‐tumor immunity. We first summarize the spleen's unique immunological architecture, then dissect how tumors hijack splenic hematopoiesis to generate immunosuppressive cell reservoirs. Finally, we critically evaluate emerging spleen‐targeted therapeutic strategies and discuss why effective immunotherapy may require intervention at this peripheral immune source.

## 2 Characteristics of the Spleen as a Peripheral Immune Organ

### 2.1 Structure and Function of the Spleen

The spleen is the largest secondary lymphoid organ, serving as a key site for immune surveillance, blood filtration, and hematopoietic reserve functions [21]. Unlike lymph nodes, it lacks afferent lymphatics and receives antigens only via the bloodstream [22, 23]. The splenic parenchyma consists of three closely coordinated regions: the white pulp (WP), the marginal zone (MZ), and the red pulp (RP). This organization forms a highly efficient system for immune surveillance and blood filtration [21, 24].

The WP consists of densely packed lymphocytes surrounding the central arterioles, with an architecture resembling that of lymph nodes. However, a key distinction is the absence of lymphatic sinuses. Consequently, during immune responses, immune cells and antigens are delivered directly to the spleen via the bloodstream rather than through lymphatic drainage [22]. The WP is functionally divided into two distinct zones: the T cell zone (TCZ) and the B cell zone (BCZ). The TCZ, also known as the periarteriolar lymphoid sheath (PALS), is enriched with high densities of T cells, where dendritic cells (DCs) interact with CD4^+^ and CD8^+^ T cells to efficiently present antigens and initiate cellular immune responses [25, 26]. The BCZ comprises B cell follicles, which can be activated during humoral immune responses to form T cell–dependent germinal centers (GCs). Within these GCs, B cells undergo somatic hypermutation and affinity maturation, ultimately generating high‐affinity antibodies [27, 28]. The structural integrity and functional maintenance of the WP are tightly regulated by specific chemokines. CCL19 and CCL21 bind to their receptor CCR7 to guide T cells and DCs into the TCZ [29]. In vivo imaging studies of lymph nodes reveal that CCR7 ligand interactions not only guide T cell homing but also stimulate the basal motility of T cells within lymphoid organs [30]. Meanwhile, CXCL13 directs B cell homing to the BCZ via CXCR5 [31], and PI3Kδ is specifically involved in CXCR5 signaling; its inhibition by idelalisib reduces migration efficiency by approximately 80% [32]. These chemokine networks play critical roles in initiating adaptive immune responses. However, in tumor conditions such as mantle cell lymphoma or hairy cell leukaemia, the immunological homeostasis of the WP is often disrupted [33]. This disruption may lead to the collapse of GC architecture, defective antigen presentation, and impaired T and B cell activation, ultimately undermining the spleen's ability to mount effective adaptive immune responses. As a result, systemic immune surveillance against malignant or infected cells could be significantly compromised, potentially facilitating tumor immune evasion and disease progression [34].

The RP comprises approximately 75% of the spleen and is the primary site for blood filtration [35]. The splenic circulation is typically divided into two patterns: closed and open circulation [36]. In the closed circulation, about 90% of blood flows directly into sinusoids and then drains into the splenic vein. In contrast, only about 10% of blood passes through the parenchyma of the RP and enters the venous sinusoids in the open circulation [37]. The inter‐endothelial slits (IES) between RP sinusoidal endothelial cells are extremely narrow (200–500 nm), forming a precise physical filtration barrier. Healthy red blood cells (RBCs) can deform and pass through, whereas senescent or abnormal RBCs are phagocytosed by red pulp macrophages (RPMs) for iron recycling [22, 23, 38]. Beyond RBC clearance, the RP is a key site for innate and effector immunity. Although adaptive responses begin in the WP, effector functions occur primarily in the RP, which harbors T cells, DCs, NK cells, and MDSCs that contribute to immune surveillance [39]. Notably, during inflammatory or tumoral challenges, MDSCs dynamically accumulate in the RP, where they suppress T cell activity and shape the adaptive immune landscape [40, 41]. Moreover, plasmablasts generated in the WP migrate to the RP in a CXCL12‐dependent manner, where they differentiate into antibody‐secreting plasma cells, enabling sustained systemic antibody production [42]. Effector CD8^+^ T cells also traffic to the RP upon activation, positioning themselves at the frontline to eliminate blood‐borne pathogens and tumor cells [43]. Thus, the RP functions not only as a mechanical filter but also as an immunologically active niche that integrates innate sensing, effector cell deployment, and humoral immunity, making it a critical hub for both homeostatic maintenance and systemic immune defense.

**FIGURE 1:**
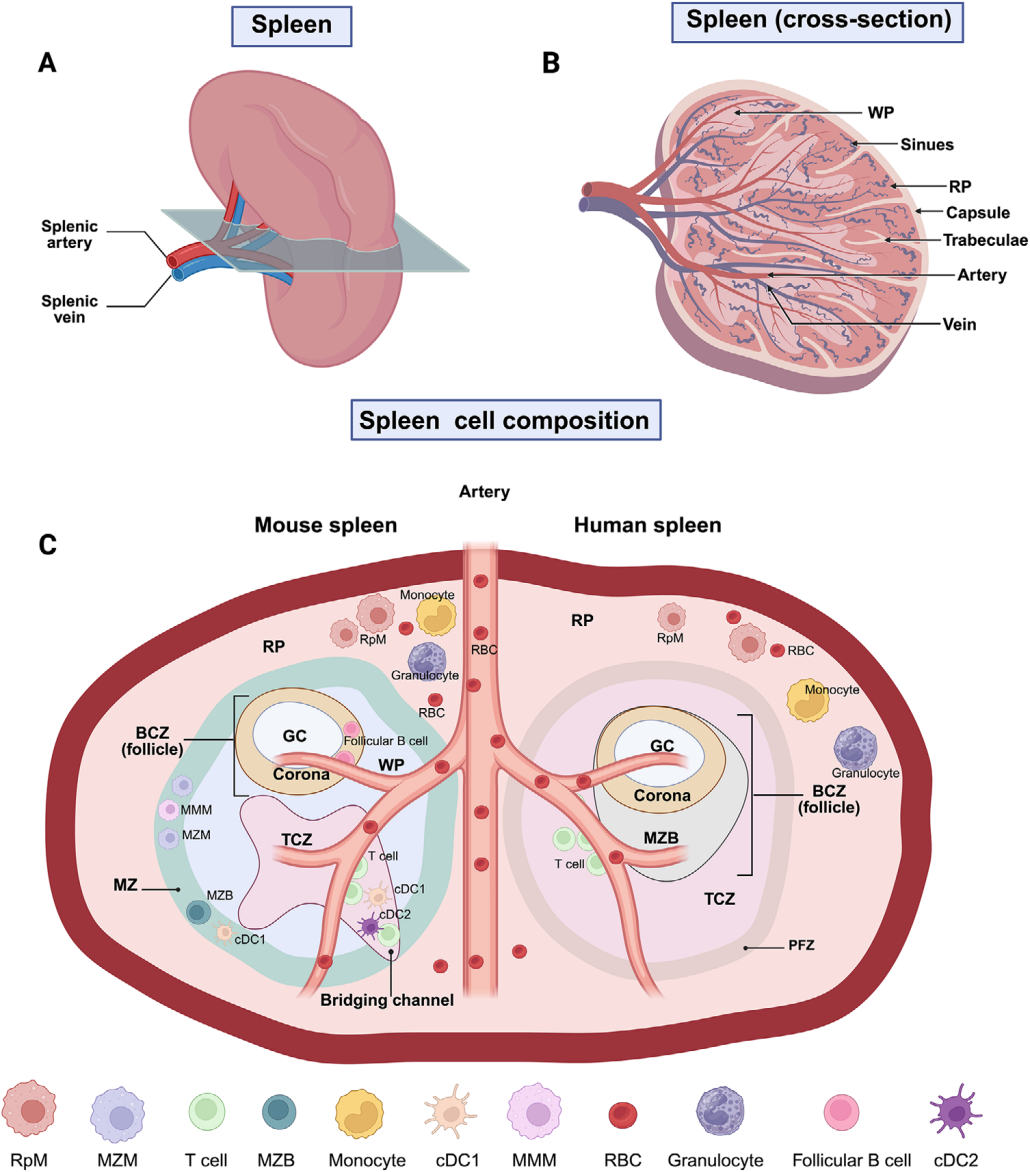
Architecture and immune cell landscape of the spleen in homeostasis. (A) Gross morphology and vascular supply of the spleen. (B) Cross‐section of the spleen showing the capsule, trabeculae, sinuses, WP, and RP. (C) Major immune cell populations localized in distinct splenic compartments. Created in https://BioRender.com.

The MZ bridges the RP and WP, capturing blood‐borne antigens and guiding immune cell trafficking [44, 45]. Rodents have a well‐defined MZ with specialized B cells and macrophages and a bridging channel for lymphocyte traffic, enabling efficient antigen capture and initiation of primary immune responses [46, 47]. In contrast, the corresponding region in the human spleen is less distinct and is commonly referred to as the perifollicular zone (PFZ) (Figure 1). It lacks clearly defined bridging channels and dense populations of marginal zone B cells (MZ B cells) and functional macrophages, resulting in reduced antigen capture efficiency and impaired lymphocyte homing, thereby weakening the initiation of adaptive immune responses [10, 48]. Under tumor burden, the structural integrity of the PFZ in the human spleen is frequently compromised, facilitating the egress of Tregs and MDSCs from this region into the peripheral circulation. This exacerbates systemic immunosuppression, promotes tumor immune escape, and ultimately limits the clinical efficacy of ICB therapy [49, 50]. Currently, most spleen‐targeted delivery systems are designed based on murine models and rely on the intact and functionally active MZ structure for efficient antigen presentation and immune activation. However, significant species differences exist between humans and mice in splenic anatomy, hemodynamic properties, and immune cell distribution, leading to markedly reduced accumulation of nanoparticles in the human PFZ region. This poses a major barrier to the clinical translation of such strategies [39, 51]. Therefore, there is an urgent need to develop precision‐targeting approaches that are specifically designed to match the structural and functional properties of the human spleen.

### 2.2 Immune Cell Populations in the Spleen

The spleen's immune function relies on a highly specialized and precisely organized repertoire of immune cells. These cells reside and interact within the unique microenvironment formed by the RP, WP, and MZ, collectively forming an efficient network for immune surveillance and response. As the largest secondary lymphoid organ in the human body, the spleen is a central site for the coordinated regulation of innate and adaptive immunity. It harbors approximately one‐third of the body's immune cells, including about one‐quarter of the body's lymphocytes, as well as substantial populations of myeloid cells such as macrophages and DCs [23, 52, 53]. Together, these constitute a highly complex reservoir and activation center for immune responses. Table 1 summarizes the types, distribution, and functions of the major immune cell populations within the spleen. The abundance and spatial organization of these immune populations provide a fertile ground for tumor‐driven reprogramming toward immunosuppressive outputs.

**TABLE 1:** Major immune cells in the spleen and their roles in tumor immunity.

Cell type	Localization	Key functions	Identification markers	Role in cancer	Refs
MZ B cells	MZ	Rapid T‐independent antibody response to blood‐borne pathogens; natural Ig production	Human: CD19^+^ CD20^+^ CD27^+^ IgM^+^ IgD^+^; mouse: CD19^+^ CD23^−^ CD21^hi^ IgM^hi^ IgD^lo^	Immunosuppressive MZBs are enriched in the head and neck squamous cell carcinoma (HNSCC) microenvironment and promote tumor progression through immune modulation; antibody responses can significantly enhance antitumor immunity and improve patient prognosis.	[47, 54, 55]
Follicular B cells	Follicles (WP)	T‐dependent humoral immunity; GC formation	Human/mouse:CD19^+^ CD20^+^ CD23^+^ CD21^int^ IgM^int^ IgD^hi^	In non‐small cell lung cancer (NSCLC) patients, a high density of follicular B cells is significantly correlated with long‐term survival; regulate CD4^+^ T cell responses via MHC‐II and CD40L, potentially suppressing antitumor immunity.	[56, 57, 58]
Naive T cells	PALS	Await antigen presentation for activation and differentiation	Human: CD3^+^ CD45RA^+^ CD45RO^−^ CCR7^+^; mouse: CD3^+^ CD44^low^ CD62L^high^ CCR7^+^	Differentiate into effector T cells, which mediate direct killing of tumor cells through the release of perforin and granzymes; tumor‐associated fibroblasts convert naïve CD4^+^ T cells into Tregs, reducing tumor‐specific T‐cell infiltration.	[59, 60]
Tregs	TCZ, RP	Maintain self‐tolerance; suppress effector T cell responses	Human/mouse: CD3^+^ CD4^+^ CD25^+^ FoxP3^+^ CD127^lo^	Suppress effector T cells in the TME, promoting immune evasion and tumor progression; analysis of splenic Treg subsets (e.g., CD161^+^ and CD26^+^) provides new insights for lymphoma prognosis and treatment.	[61, 62]
Conventional DCs (cDC1)	TCZ, MZ	presentation to CD8^+^ T cells; critical for anti‐tumor immunity	Human: CD141^hi^ XCR1^+^ CLEC9A^+^; mouse: CD8α^+^ XCR1^+^ CLEC9A^+^ CD103^+^	In the murine melanoma model, intranasally administered vaccines can specifically target CD103^+^ migratory cDC1, present tumor antigens to cytotoxic T cells; the baseline level of cDC1 in tumors is significantly higher in patients sensitive to PD‐1 therapy compared to those who are resistant.	[60, 63]
Conventional DCs (cDC2)	TCZ, MZ	Activate CD4^+^ T cells; drive Th2/Th17 responses	Human: CD1c^+^ SIRPα^+^ CD11c^+^; mouse: CD11b^+^ SIRPα^+^ CD11c^+^	cDC2‐activated CD4^+^ T cell response correlates with improved survival in lung and breast cancer patients; baseline quantity of cDC2 in tumor‐draining lymph nodes positively correlates with patients' pathological response to PD‐1 therapy.	[64, 65, 66]
Tolerogenic DCs (tolDCs)	MZ, RP, PALS	Induce T cell anergy and apoptosis; promote differentiation and expansion of Tregs	Human: ILT3/4^+^, DC‐SIGN^+^, CLEC4A^+^; Mouse: CD8α^+^, CD301b^+^	Accumulate in the spleen during progression of various cancers (e.g., pancreatic, hepatocellular, breast); act as central mediators of systemic immunosuppression, facilitating tumor immune escape; preclinical studies show that targeting splenic tolDCs enhances anti‐tumor immunity and immunotherapy efficacy	[67, 68, 69, 70]
RPMs	RP	Clear senescent RBCs; iron recycling	Human: F4/80^hi^ CD11b^lo^ VCAM1^+^CD163^+^; Mouse: F4/80^hi^ CD11b^lo^ VCAM1^+^	In chronic myeloid leukemia (CML), it promotes the accumulation of leukemia stem cells and disease progression by shaping the microenvironment. In colorectal cancer (CRC), it drives cancer progression by modulating metabolic processes within the TME. In oral squamous cell carcinoma (OSCC), it exhibits tumor‐promoting properties through interactions with cancer cells.	[71, 72, 73]
Marginal zone macrophages (MZMs)	MZ	Capture blood‐borne antigens and pathogens	Human/Mouse: CD68^+^ MARCO^+^	In prostate cancer patients, it promotes tumor progression via lipid dysregulation and IL‑1β signaling, linked to shorter progression‑free survival (PFS); in a mouse pancreatic ductal adenocarcinoma (PDAC), depletion of MZM reduces tumor volume by approximately 50%.	[74, 75]
Metallophilic marginal zone macrophages (MMMs)	Inner marginal sinus	Regulate lymphocyte entry into WP	Human/Mouse: CD68^+^ CD169^+^ (Siglec‐1)	In glioma models, MMM promotes T/NK cell recruitment via CXCL9/10, enhancing anti‐tumor immunity; high CD169^+^ macrophage infiltration correlates with better survival in CRC patients.	[74, 76]
MDSCs	RP, circulation	Suppresses T cell and NK cell function via ARG1, iNOS, and ROS, while producing IL‐10 and TGF‐β.	Human: CD11b^+^ CD33^+^ HLA‐DR^lo/neg^ (PMN‐MDSC: CD14^−^ CD15^+^; M‐MDSC: CD14^+^ CD15^−^); Mouse: CD11b^+^ Gr1^+^ (PMN‐MDSCs: CD11b^+^ Ly6G^+^ Ly6Cˡ°; M‐MDSCs: CD11b^+^ Ly6G^−^ Ly6C^hi^)	High peripheral M‑MDSC levels correlate with reduced ICB efficacy, shortening PFS by 40%–50%; in non‑Hodgkin lymphoma patients, >50% reduction in circulating MDSCs triples objective response rate.	[77, 78, 79]

### 2.3 Immune Activation and Immune Suppression in the Spleen

As a pivotal immune hub, the spleen orchestrates both innate and adaptive immunity. Macrophages and DCs in the RP and MZ rapidly eliminate pathogens and cellular debris, triggering the first line of defense [23, 44]. This function is critically extended to anti‐tumor immunity, where the spleen serves as a primary site for initiating systemic responses. It acts as a site where naive T cells are primed by tumor‐associated antigens, often presented by DCs. Within the WP, these T cells undergo clonal expansion and differentiate into cytotoxic T lymphocytes (CTLs) and helper T cells, generating a large army of tumor‐specific effector cells [80, 81]. For example, in the B16‑OVA melanoma model, spleen‑targeted mRNA vaccines (mRNA‑sLNPs) optimized with ionizable lipids increased splenic mRNA translation efficiency by 3.2‑fold and markedly enhanced antigen‑specific CD8^+^ T‑cell responses (reaching 4.5‑fold higher than the control) [82]. Splenectomized mice completely lost this protective effect, confirming the spleen's essential role in immune initiation [83]. Concurrently, in the BCZs, antigen‐specific B cells are activated and differentiate within GCs, ultimately producing high‐affinity antibodies [84, 85]. Furthermore, the spleen serves as a major reservoir for memory T and B cells, supporting long‐lasting immunological memory against tumors. In the B16F10 melanoma model, inducing tumor cell pyroptosis triggers robust immune memory that protects against lymph node metastases, as confirmed by resistance to secondary tumor challenge [86]. Similarly, combination therapy with chemotherapy and immunotherapy enhances the pool of effector memory T cells in the spleen, leading to significantly reduced tumor recurrence and metastasis [87]. Thus, through its unique architecture, the spleen coordinates multiple aspects of the immune attack, serving as a critical hub for initiating a systemic immune response against cancer.

However, the spleen is not only a central site for initiating immune responses but also a key regulatory organ for immune suppression. During tumor progression, the spleen actively contributes to systemic immunosuppression through the coordinated activity of myeloid cell populations in the MZ. It becomes a major reservoir for the expansion and recruitment of CD11b^+^Gr‐1^+^Ly6C^hi^ MDSCs [40]. These cells disrupt the normal architecture of the MZ and establish close contact with CD8^+^ T cells. They can phagocytose and cross‐present tumor antigens but simultaneously release ROS and NO, and deplete arginine, collectively leading to the functional inactivation of tumor‐specific CD8^+^ T cells [23, 40]. MDSC depletion using anti‑Gr‑1 antibodies in D5 melanoma mice boosted tumor‑draining lymph node IFN‑γ‑secreting T cells, with a 2.3‑fold increase in IFN‑γ secretion [88]. In addition to MDSCs, recent studies have identified a novel population of immunosuppressive IDO1^+^ tolerogenic DCs in the spleens of cancer patients. In PDAC, these cells accumulate in the spleen and mediate broad immune dysregulation through extensive crosstalk with innate and adaptive immune cells, potentially via AHR, IDO‐1, CCL19, and related signaling pathways [68]. Moreover, MZMs and CD169^+^ MMMs amplify immunosuppression by clearing self‐antigens and secreting CCL22, a macrophage‐derived immunosuppressive chemokine [89]. CCL22 recruits FoxP3^+^ Tregs into the spleen via the CCR4 receptor, a mechanism known as the CCL22/CCR4 axis. In hepatocellular carcinoma (HCC), Treg infiltration driven by this axis is associated with poor prognosis, highlighting its clinical relevance [90]. Once enriched, Tregs produce anti‐inflammatory cytokines such as TGF‐β and IL‐10, which further dampen effector immune responses and establish a self‐reinforcing immunosuppressive microenvironment [91, 92]. Together, these mechanisms underscore the spleen's dual capacity as both an immune activator and a potent hub for immunosuppression, highlighting its critical influence on the balance between anti‐tumor immunity and immune escape.

## 3 Tumor‐Induced Spleen Remodeling: A Driver of Systemic Myeloid Expansion

Analogous to TME remodeling, tumors actively reshape the spleen into a pro‐tumorigenic immune compartment. A central manifestation of this process is the induction of pathological EMH, which reprograms splenic hematopoiesis toward the sustained production of immunosuppressive myeloid populations.

### 3.1 The Spleen as a Primary Site of Tumor‐Elicited EMH

Under physiological conditions, hematopoiesis primarily occurs in the bone marrow. However, the spleen retains its fetal capacity for hematopoiesis and becomes a major site of EMH in pathological conditions such as chronic inflammation, myelofibrosis, and hematopoietic disorders, reactivating blood cell production [93, 94, 95]. In cancer, normal bone marrow hematopoiesis is often disrupted, shifting toward myeloid‐biased differentiation with expanded myeloid populations and suppressed erythroid and lymphoid lineages [96, 97, 98]. Under tumor‐induced stress, the spleen is activated as a key site of EMH, supporting the proliferation and differentiation of hematopoietic stem and progenitor cells (HSPCs) to meet increased demand for blood cells [41, 99].

Beyond simply reactivating hematopoietic output, tumor‐induced splenic EMH also drives profound structural and functional alterations within the splenic parenchyma. The massive accumulation of HSPCs and myeloid cells from pathological EMH exerts direct physical pressure on the WP, disrupting the structural integrity of its TCZs and BCZs and the chemokine gradients that sustain WP compartmentalization and immune cell crosstalk [52, 100]. Moreover, the robust expansion of immunosuppressive myeloid cells in the RP impairs MZ antigen presentation through both structural disruption and soluble mediator release. RP‐derived myeloid cells infiltrate the MZ, disrupting the architectural organization of antigen‐capturing macrophages and MZ B cells, and hinder the trafficking of blood‐borne antigens to MZ DCs [97]. In addition, these cells secrete high levels of immunosuppressive factors such as IL‐6, TGF‐β, ROS, and ARG1, and upregulate PD‐L1 expression within the MZ microenvironment [101, 102]. Importantly, tumor‐induced splenic EMH extends beyond compensating for impaired bone marrow function. It actively generates immunosuppressive myeloid cells that promote systemic tumor immune escape, positioning the spleen as a central hub of cancer‐associated immunosuppression [41].

### 3.2 Mechanisms of Tumor‐Induced Splenic EMH

Splenic EMH is a hallmark of tumor‐induced systemic remodeling, driven by TDFs that mobilize HSPCs, chemokine axis‐mediated migration and retention of HSPC, and the local “educational” role of the splenic stromal microenvironment that shapes myeloid differentiation.

#### 3.2.1 Remote Regulation of Splenic Hematopoiesis by Tumor‐Derived Factors

Tumor cells establish a systemic environment that skews hematopoietic differentiation by secreting a variety of soluble factors, directly driving the activation of splenic EMH and myeloid‐biased differentiation [103]. Colony‐stimulating factors (CSF), particularly granulocyte CSF (G‐CSF), GM‐CSF, and macrophage CSF (M‐CSF), are key mediators of pathological myeloid expansion [104, 105]. In the murine 4T1 breast cancer model, daily administration of recombinant G‐CSF increases the frequency of splenic Gr‐1^hi^CD11b^+^ MDSCs from a baseline of 12.3% ± 2.1% to 38.7% ± 4.5%, while enhancing mobilization of bone marrow CD34^+^ HSPCs by 3.2‐fold [106]. Genetic ablation of the G‐CSF receptor (Csf3r^−^/^−^) in tumor‐bearing mice reduces splenic MDSC expansion by 76%, confirming its central role in Gr‐1^hi^ subset accumulation [107]. In contrast, deletion of the GM‐CSF receptor (Csf2rb^−^/^−^) predominantly affects the Gr‐1^int/low^ MDSC population, reducing it by 58% [108]. Clinically, in a cohort of 45 CRC patients, serum GM‐CSF levels positively correlate with circulating CD14^+^HLA‐DR^low^ MDSC counts [109], underscoring the translational relevance of these pathways. Beyond CSFs, VEGF and M‐CSF further skew splenic hematopoiesis toward immunosuppressive lineages. In a myocardial infarction mouse model, splenic VEGF levels rise 3.5‐fold, coinciding with a 62% reduction in splenic CD3^+^ T cells [110]. Mechanistically, VEGF signaling via VEGFR2 directly suppresses IL‐7 production by splenic stromal cells, a critical cytokine for lymphopoiesis [111]. Meanwhile, M‐CSF promotes myeloid bias by polarizing splenic macrophages toward an M2 phenotype, increasing the proportion of CD206^+^ cells from 15% to 54% [112], which in turn further supports an immunosuppressive microenvironment.

The inflammatory milieu further modulates this process through coordinated signaling networks. Inflammatory cytokines such as IL‐6 and IL‐1β form a synergistic network with CSFs, collectively shaping the microenvironment for HSPC differentiation into MDSCs [23, 41]. In acute myeloid leukemia (AML), IL‐6 is predominantly secreted by mesenchymal stromal cells (MSCs), and elevated IL‐6 levels are strongly associated with poor prognosis. Selective deletion of Il6 in MSCs significantly reduces chemoresistance in AML‐bearing mice [113]. Similarly, IL‐1 signaling plays a critical role in pathological hematopoiesis. Treatment with IL‐1 receptor antagonist (IL‐1Ra) reduces CD34^+^ progenitor cell expansion by 40% in AML patients, and low circulating IL‐1Ra levels correlate directly with reduced overall survival [114]. Importantly, non‐cytokine molecules also contribute to splenic EMH. Tumor‐derived angiotensin II (AngII) promotes retention of HSPCs in the splenic RP by inhibiting sphingosine‐1‐phosphate receptor 1 (S1P1) signaling. In a myocardial infarction model, 7‐day treatment with angiotensin‐converting enzyme (ACE) inhibitors promotes HSPC retention within the marrow niche and reduces splenic seeding [115]. This demonstrates that AngII signaling is not only a key regulator of HSPC trafficking but also a pharmacologically targetable pathway in cancer‐ and inflammation‐associated hematopoiesis [116]. Together, these systemic signals function as a “remote control” system, priming distant hematopoietic organs for immunosuppressive reprogramming even before HSPCs reach the spleen.

#### 3.2.2 Directed Migration of HSPCs From the Bone Marrow to the Spleen

The successful initiation of splenic EMH depends on the precise trafficking of HSPCs from the bone marrow to the spleen, a process governed by chemokine‐guided homing mechanisms. Tumor‐induced elevation of circulating CCL2 facilitates the egress of CCR2‐expressing HSPCs, as evidenced by intravital imaging showing a 4.3‐fold increase in HSPC transmigration across bone marrow sinusoids upon CCL2 stimulation [117]. At the same time, local production of CCL2 in the spleen by VE‐cadherin^+^ vascular endothelial cells and nestin^+^ MSCs establishes a chemotactic gradient that directs HSPC recruitment and colonization of the splenic microenvironment [40, 41]. In Apc^(Min/+) intestinal tumor models, CCR2 knockout reduced splenic HSPC accumulation by 62% compared to wild‐type controls, while CCL2 neutralization decreased EMH‐derived myeloid cells by 55% in breast cancer‐bearing mice [118]. Thus, the CCL2/CCR2 axis is indispensable for directing HSPC trafficking and colonization in the spleen during tumorigenesis.

Additional chemokine axes also contribute to HSPC mobilization. For example, CXCL12 (SDF‐1)/CXCR4 signaling, which regulates bone marrow retention, is dynamically modulated during cancer progression. Reduced CXCL12 expression in the bone marrow, coupled with increased levels in the spleen, creates a redistributive gradient that favors HSPC mobilization from the marrow and homing to the spleen [119]. Moreover, tumor‐associated inflammation enhances vascular permeability in the spleen, facilitating transendothelial migration of circulating HSPCs [23]. This spatial reprogramming of hematopoietic trafficking ensures a continuous supply of precursor cells to support the expanding demand for myeloid output in the spleen. Thus, the directed migration of HSPCs represents a critical checkpoint linking systemic tumor signaling to localized EMH activation.

#### 3.2.3 Local Reprogramming by the Splenic Stromal Microenvironment

Once HSPCs home to the spleen, their retention, expansion, and functional fate are tightly regulated by the local stromal microenvironment, which is dynamically remodeled in response to tumor‐derived signals. RPMs play a structural and signaling role by expressing vascular cell adhesion molecule‐1 (VCAM‐1), which anchors HSPCs via VLA‐4 (α4β1 integrin) interactions. Disruption of macrophage function or specific silencing of Vcam1 markedly impairs splenic EMH, highlighting the essential scaffolding role of RPMs in maintaining the EMH niche [120]. Beyond physical retention, splenic stromal cells also play an instructive role in HSPC programming. Upon receiving TDF signals, these cells secrete factors like IL‐6 that synergize with endogenous GM‐CSF to functionally “educate” HSPCs, driving their differentiation toward immunosuppressive myeloid lineages [41]. Furthermore, platelet‐derived growth factor (PDGF‐BB) and VEGF stimulate stromal cells to produce erythropoietin (EPO). While EPO promotes erythropoiesis, it also interferes with T cell migration by inhibiting stromal production of the T cell chemokines CCL19/CCL21, thereby creating an immunosuppressive microenvironment [121, 122, 123].

### 3.3 Splenic EMH Generates Immunosuppressive Erythroid‐Derived Myeloid Cells

**FIGURE 2:**
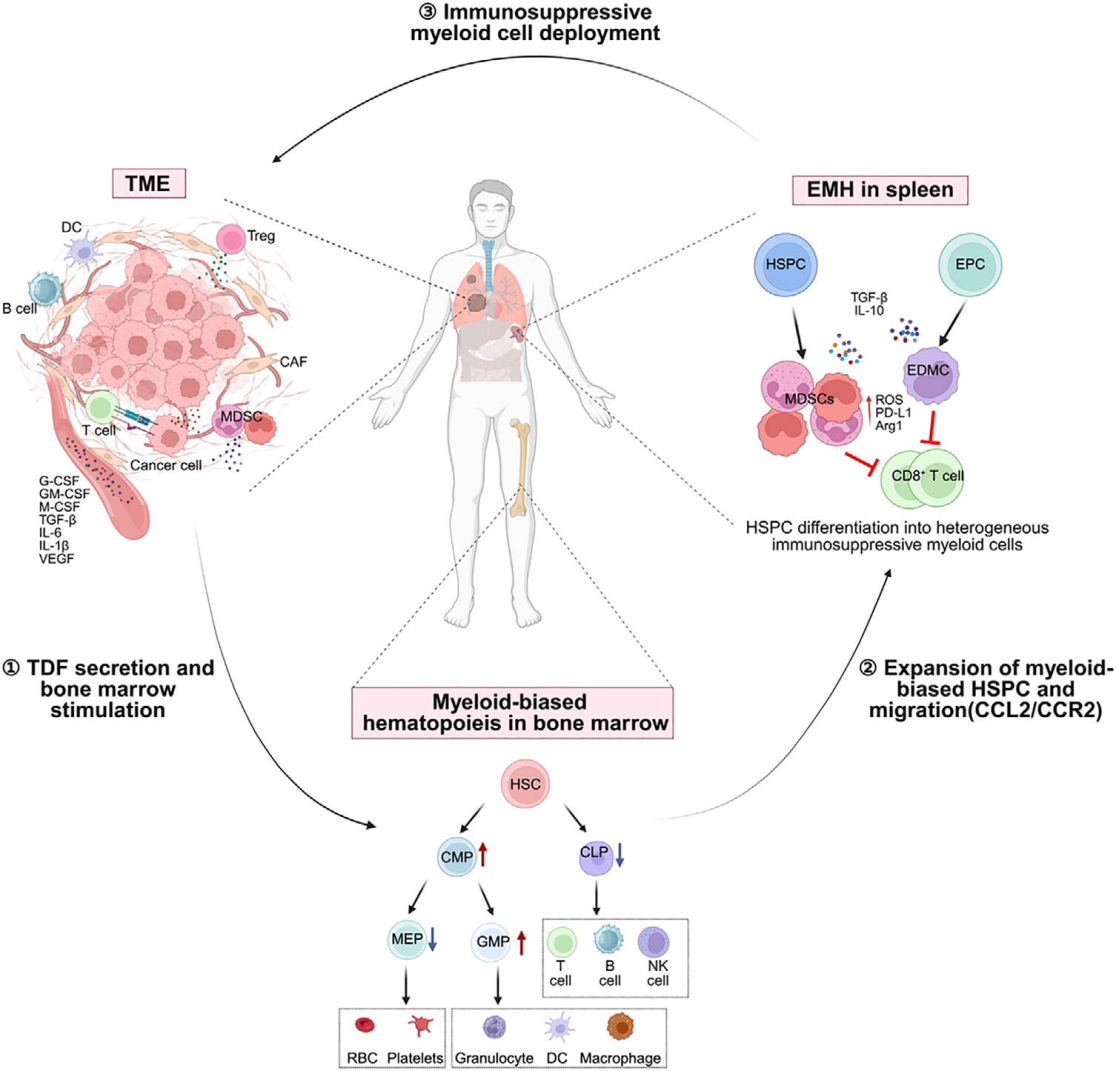
Cancer‐associated splenic remodeling is mediated by tumor‐derived soluble factors. TDFs (e.g., G‐CSF, IL‐6, VEGF) drive splenic remodeling by promoting myeloid‐biased hematopoiesis and HSPC migration from bone marrow to spleen via CCL2/CCR2. This establishes a niche for EMH, expanding immunosuppressive myeloid cells that support tumor progression. Created in https://BioRender.com.

Beyond classical myeloid differentiation, splenic EMH under tumor conditions also exhibits aberrant erythropoiesis. Studies show that a large number of erythroid progenitor cells (EPCs) accumulate in the spleen in response to tumor‐induced anemia [124]. However, systemic GM‐CSF, largely derived from the TME, blocks the maturation of EPCs into mature RBCs and redirects their differentiation toward a myeloid lineage, giving rise to immunosuppressive erythroid‐derived myeloid cells (EDMCs) [101]. EDMCs are distinct from classical MDSCs in origin, phenotype, and suppressive mechanisms. While classical MDSCs derive from the myeloid lineage of HSPCs, with PMN‐MDSCs and M‐MDSCs both differentiating from granulocyte‐macrophage progenitors (GMPs) in the bone marrow and spleen under tumor‐derived cytokine stimulation [23, 41], EDMCs originate from EPCs, a non‐myeloid hematopoietic lineage whose normal RBC maturation is blocked by tumor‐derived GM‐CSF. This leads to their aberrant myeloid reprogramming and the acquisition of an immunosuppressive phenotype in the tumor‐induced splenic microenvironment. Phenotypically, classical PMN‐MDSCs are CD11b^+^Ly6G^+^Ly6C^low^ in mice and CD11b^+^CD15^+^CD14^−^HLA‐DR^lo/neg^ in humans, and M‐MDSCs are CD11b^+^Ly6G^−^Ly6C^hi^ in mice and CD11b^+^CD14^+^CD15^−^HLA‐DR^lo/neg^ in humans [77, 78, 79]. By contrast, EDMCs lack these typical MDSC markers and are characterized by CD71^+^Ter119^+^ in murine models and CD71^+^glycophorin A^+^ (GPA^+^) in humans, retaining core erythroid lineage surface markers while acquiring immunosuppressive functional features [125]. Mechanistically, both cell populations inhibit T cell function via ARG1 and ROS production, but EDMCs exert far more potent suppression by highly upregulating PD‐L1, a molecule only weakly expressed in classical MDSCs, with the PD‐L1/PD‐1 signaling axis acting as their dominant mediator of T cell exhaustion and inactivation [101]. Classical MDSCs additionally rely on iNOS and IDO for immunosuppression, pathways that are not primary for EDMCs [102]. Clinically, intratumoral infiltration of EDMCs is strongly associated with T cell exhaustion and poor patient prognosis, and their predictive power for survival outcomes exceeds that of conventional MDSCs and Tregs [101, 126]. This finding reveals a previously unrecognized plasticity in hematopoietic lineage commitment and identifies EDMCs as a key mediator of spleen‐derived immunosuppression in cancer (Figure 2). Collectively, these findings support a model in which tumors remotely program splenic hematopoiesis through systemic priming, directed progenitor trafficking, and local stromal education, thereby converting the spleen into a self‐sustaining source of immunosuppressive cells.

## 4 The Role of Spleen in Systemic Immunosuppression and Immunotherapy Resistance

### 4.1 The Spleen Sustains the Production of Immunosuppressive Myeloid Cells

During tumor progression, the spleen functions as a persistent and active site for immunosuppressive cells, continuously generating and exporting MDSCs [127, 128]. In response to TME‐induced emergency myelopoiesis, immature myeloid cells (IMCs) migrate to the spleen, the primary site of EMH, where they expand and differentiate into functionally activated MDSCs. This positions the spleen as a central hub for MDSC accumulation and activation [127]. In cancer patients, the frequency of granulocytic MDSCs (PMN‐MDSCs, CD14^−^CD15^+^) in the spleen is significantly elevated and correlates with poor prognosis. Functional experiments have confirmed for the first time that MDSCs isolated from the human spleen can effectively suppress T cell responses, demonstrating their potent immunosuppressive activity [127].

These observations are supported by extensive preclinical evidence. In multiple tumor‐bearing mouse models, including the 4T1 breast cancer model, splenic EMH has been identified as the principal source of tumor‐infiltrating MDSCs prior to their entry into the TME [128]. Lineage tracing approaches, including in vivo BrdU incorporation and CFSE‐based tracking, reveal that myeloid proliferation and retention are markedly higher in the spleen than in the bone marrow during tumor progression [129, 130, 131]. MDSCs preferentially accumulate in the subcapsular RP and subsequently infiltrate both the RP and MZ, forming a dynamic pool that can rapidly re‐enter the circulation and traffic to tumor sites [130, 131]. This splenic expansion is characterized by robust local proliferation of MDSCs, a feature that is largely restricted to the tumor‐bearing state [131].

### 4.2 The Spleen Shapes an Immunosuppressive TME

The immunosuppressive role of the spleen extends beyond myeloid cell production and influences anti‐tumor immunity through both local interactions within splenic niches and the systemic supply of suppressive effector cells to the TME. For example, within the spleen, MDSCs exert direct and potent suppressive effects on adaptive immunity [132]. In patients with gastric and pancreatic cancers, the spleen is enriched within highly suppressive PMN‐MDSCs characterized by elevated expression of PD‐L1, LOX‐1, and phosphorylated STAT3, together with increased production of ROS. Histological analyses show that these cells physically interact with T cells in the WP and PALS, leading to marked inhibition of T cell proliferation and IFN‐γ production [133, 134]. Importantly, the frequency of these splenic PMN‐MDSCs inversely correlates with overall survival, underscoring their clinical relevance [135].

Upon their mobilization to tumor sites, splenic‐derived MDSCs become dominant regulators of immune evasion within the TME. They suppress CD8^+^ T cell function through multiple mechanisms, including depletion of L‐arginine via ARG1, NO production through iNOS, and secretion of immunosuppressive cytokines such as TGF‐β. In parallel, MDSCs promote the expansion and stabilization of Tregs, reinforcing a suppressive cellular network within the tumor [102]. The collaborative interaction between MDSCs and Tregs in the TME fosters a profoundly immunosuppressive milieu, and high infiltration of these cells is negatively correlated with the efficacy of ICB [136, 137]. In addition, MDSCs attenuate innate anti‐tumor immunity by impairing NK cell activation and cytotoxic function through contact‐dependent mechanisms [138].

**FIGURE 3:**
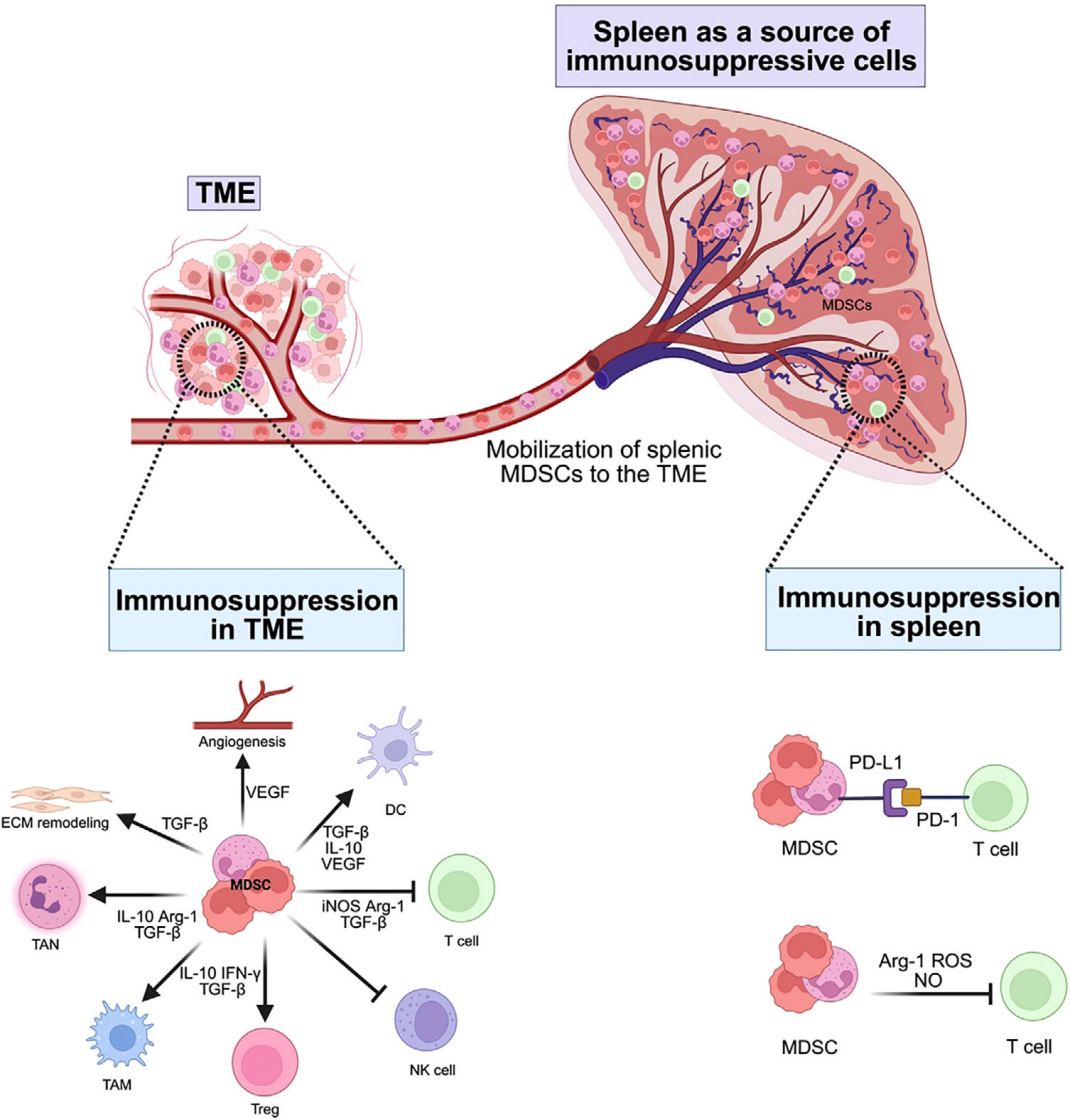
Systemic immunosuppression mediated by splenic myelopoiesis during tumor progression. As tumors progress, the spleen expands immunosuppressive MDSCs via TDFs. These MDSCs express PD‐L1, inhibiting T cell activity in the spleen, and migrate to tumors, where they secrete IL‐10, IL‐6, TGF‐β, and ARG1, suppressing T and NK cell function and promoting Tregs, TAMs, and TANs accumulation. Created in https://BioRender.com.

Beyond MDSCs, the spleen also serves as a continuous source of other immunosuppressive cells such as tumor‐associated macrophages (TAMs) and tumor‐associated neutrophils (TANs). Newly generated CX3CR1^int^Ly6C^hi^ monocytes in the spleen continuously migrate to tumor sites in a CCR2‐dependent manner and differentiate into TAMs, which promote tumor progression [97, 116]. In lung adenocarcinoma models, hematopoietic progenitors in the splenic RP establish a proliferative niche, continuously generating monocyte and granulocyte precursors that feed the TAM and TAN pools in growing tumors, further sustaining an immunosuppressive TME (Figure 3) [97]. Together, these findings position the spleen as a central contributor to systemic and intratumoral immunosuppression, acting both as a site for the generation and conditioning of suppressive immune cells and as a sustained source of these cells to the TME.

### 4.3 Splenic Remodeling Drives Resistance to ICB

Tumor‐induced splenic remodeling is increasingly recognized not only in preclinical models but also as a clinically relevant phenomenon in cancer patients [103]. Structural and immunological changes in the spleen, including splenomegaly and altered cellular architecture, serve as measurable indicators of systemic immunosuppression and poor therapeutic response. For example, myeloproliferative neoplasms (MPNs) such as primary myelofibrosis (PMF) are often accompanied by splenic EMH, which leads to splenomegaly and increases the risk of thrombosis as well as hepatic and pulmonary complications [139]. In HCC cases, changes in spleen volume are associated with the prognosis of cancer patients undergoing ICB therapies [140]. Beyond size, the spleen also undergoes significant structural changes in cancer patients. In CML, leukemia stem cells preferentially accumulate in the RP region, in proximity to RPMs, and promote disease progression through RPM‐mediated cell cycle regulation [141]. In splenic marginal zone lymphoma (SMZL), all patients exhibit hyperplasia of splenic MZ B cells [142, 143].

Accumulating evidence highlights the spleen as a key determinant of resistance to ICB therapies, including anti‐PD‐1 and anti‐CTLA‐4 antibodies, particularly in cancers such as HCC, CRC, and melanoma [144, 145, 146]. Peripheral MDSC dynamics reflect splenic activity and strongly predict ICB outcomes. Elevated baseline levels of circulating M‐MDSCs and PMN‐MDSCs are consistently associated with poor response across multiple cancer types. In NSCLC, patients with M‐MDSC levels above the median exhibit significantly shorter PFS and overall survival (OS) [147]. Similarly, in metastatic melanoma, high baseline frequencies of circulating M‐MDSCs are associated with poor clinical response to ICB, although those who show a rapid reduction in MDSC levels during treatment often achieve better outcomes, suggesting that dynamic changes in MDSCs may serve as an early biomarker of therapeutic response [78]. In clear cell renal cell carcinoma (ccRCC), both baseline M‐MDSC levels and their proportion within the total MDSC pool predict ICB response, with non‐responders showing no significant decline in MDSCs after therapy [148]. In CRC, tumor‐infiltrating CCR2^+^ PMN‐MDSCs suppress CD8^+^ T cell activity and drive disease progression, and preclinical studies demonstrate that blocking their recruitment can reduce tumor burden [149]. In prostate cancer, elevated levels of circulating MDSCs correlate with worse OS, further supporting their potential as prognostic markers and therapeutic targets [150]. Collectively, these clinical findings highlight the systemic impact of splenic remodeling. By maintaining a sustained pool of immunosuppressive myeloid cells, the spleen plays an active role in shaping an immune environment that resists checkpoint inhibition.

## 5 Targeting the Spleen for Cancer Therapy

### 5.1 The Spleen as an Immunotherapy Target

Splenic targeting in immunotherapy has key advantages over lymph node targeting. As the largest secondary lymphoid organ, the spleen contains a higher density of antigen‐presenting cells (APCs) and lymphocytes [22]. Its unique microvascular architecture and slow blood flow enable efficient antigen capture by APCs [21, 151]. While subcutaneous vaccines depend on slow APC migration to lymph nodes [152], intravenously delivered spleen‐targeting nanovaccines are taken up directly by splenic APCs, leading to rapid T and B cell activation and stronger, more durable immune responses [152].

Despite its promise as an immunotherapy target, direct splenic modulation in immunotherapy faces major challenges. Its dense endothelial cell network and robust phagocytic system rapidly sequester and clear foreign substances, including therapeutic agents, making precise drug delivery technologically difficult [153, 154]. The spleen also has dual roles in immunity. It can support anti‐tumor responses while simultaneously promoting immunosuppression. These suppressive effects persist even after splenectomy. In some tumor models, removing the spleen accelerates tumor growth [155, 156]. This may result from the loss of splenic anti‐tumor immune cells, such as NK cells and effector T cells, and from impaired immune surveillance. Therefore, the optimal strategy is not splenectomy, but targeted modulation of splenic immunity. This approach aims to suppress immunosuppressive activity while preserving and enhancing the spleen's anti‐tumor immune functions. Table 2 summarizes spleen‐targeting therapeutic strategies.

**TABLE 2:** Spleen‐targeting therapeutic strategies.

Strategy	Clinical stage	Safety profile	Scalability	Targeting precision	Key limitations	Disease context	Refs.
**Splenectomy**	Clinical use	High risk (OPSI, sepsis)	High	None (organ removal)	Irreversible immunosuppression; increased infection risk	Hematologic malignancies (e.g., CLL); hypersplenism, trauma	[157, 158, 159]
**Small molecule inhibitors (e.g., STAT3i, JAKi)**	Phase II–III trials	Moderate (off‐target toxicity)	High	Low (systemic exposure)	Chronic toxicity; compensatory pathways	Metastatic cancer; chronic inflammation	[160, 161, 162]
**Nanoparticle‐based vaccines**	Phase I–II (e.g., RNA‐LPX)	Favorable (biodegradable)	Moderate (batch variability)	High (size/charge‐dependent)	Liver/lung clearance; limited payload	Cancer immunotherapy; infectious diseases	[163, 164]
**Virus‐based vectors (e.g., AAV, lentivirus)**	Early‐phase gene therapy	High risk (immunogenicity, mutagenesis)	Low (complex manufacturing)	High (tropism engineering)	Pre‐existing immunity; safety concerns	Gene therapy; vaccine development	[165, 166]
**RBC‐hitchhiking**	Preclinical to translational	Favorable (biomimetic, low toxicity)	Low–Moderate (ex vivo processing)	Very high (natural clearance)	Short half‐life; low payload; scalability issues	Cancer immunotherapy; autoimmune diseases; targeted immunosuppression	[167, 168, 169]
**Neutrophil‐mediated delivery**	Preclinical (mouse models)	Moderate (inflammatory risk)	Very low (cell manipulation)	High (chemotaxis‐driven)	Short lifespan; difficult expansion	Metastatic cancer; acute inflammation	[52, 170]

### 5.2 Splenectomy

Splenectomy offers a key perspective for studying the spleen's role in tumor immunity. Experimental studies have shown that in mouse models of cancers such as HCC, splenectomy effectively suppresses primary tumor growth and metastasis, and significantly prolongs the overall survival of tumor‐bearing mice [157]. This anti‐tumor effect is closely associated with a marked reduction in MDSCs both systemically and within the TME, confirming the spleen as a major source of these key immunosuppressive cells [158, 159]. The apparent contradiction between tumor‐promoting and tumor‐suppressing effects of splenectomy across different models reflects the context‐dependent duality of splenic immunity. In early‐stage or immunologically active tumors, the spleen supports anti‐tumor responses through NK cells and effector T cells. Its removal impairs immune surveillance and accelerates tumor growth. In contrast, in advanced or chronic inflammatory cancers, the spleen becomes a dominant reservoir for immunosuppressive MDSCs generated via EMH, and splenectomy alleviates systemic immunosuppression and enhances anti‐tumor immunity. This dynamic shift highlights the spleen's functional evolution during tumor progression, transitioning from a site of immune activation to a central hub of immunosuppression. Despite these mechanistic insights, the clinical application of splenectomy is rarely used in cancer treatment due to its risks and the availability of better options [171]. The procedure impairs immune function, increasing patient susceptibility to encapsulated bacteria such as Streptococcus pneumoniae [172, 173, 174]. This can lead to overwhelming post‐splenectomy infection (OPSI), a life‐threatening condition with high mortality [175, 176, 177]. Moreover, the spleen may also support anti‑tumor immunity, and splenectomy could impair tumor control [155, 156]. Currently, splenectomy is mainly reserved for a limited number of cases where the spleen is directly invaded by tumor or its removal is necessary for surgical radicality, as in some advanced ovarian, pancreatic, or gastric cancers [178, 179]. Its independent effect on long‑term prognosis, especially under immunotherapy, remains unclear. More systematic clinical and immunological data are needed to define the spleen's precise role in human cancer progression [171, 179].

### 5.3 Targeting Tumor‐Induced Splenic EMH

Given the pivotal role of splenic EMH in tumor‐mediated immunosuppression, developing specific targeting strategies is of great importance. Current research primarily focuses on selectively eliminating pro‐tumorigenic myeloid progenitor cells in the spleen and interfering with the recruitment and retention of HSPCs to the spleen. Selective depletion strategies can target splenic myeloid progenitors without affecting the bone marrow. Low‐dose 5‐fluorouracil (5‐FU) preferentially reduces the expansion of lineage‐restricted progenitor cells in the spleen. This restores anti‐tumor immunity and enhances the efficacy of adoptive T cell therapy [40]. Similarly, low‐dose c‐Kit inhibitors can specifically induce apoptosis of early upstream HSPCs in the spleen and reduce their GM‐CSF expression. These effects impair PMN‐MDSC function and enhance the response to ICB [41].

Concerning the interference with HSPC homing and retention, the CCL2/CCR2 axis has emerged as a key target due to its central role in mediating the migration of HSPCs from the bone marrow to the spleen [40, 180]. Several CCR2 inhibitors (e.g., CCX872‐B, PF‐04136309) have already entered clinical trials to evaluate their efficacy in treating solid tumors [181]. Carlumab (CNTO888), a CCL2 neutralizing antibody, showed good tolerability and transient CCL2 suppression in a Phase I trial, with some patients achieving stable disease [182]. However, in a Phase II study in metastatic prostate cancer, it yielded no objective responses and limited disease control, with only 34% of patients maintaining stable disease beyond 3 months [183]. Preclinically, the CCR2 antagonist CCX872 improved survival in glioma models, especially when combined with immunotherapy, and this benefit was associated with reduced tumor‐infiltrating MDSCs [184]. CCR2/5 inhibitors such as BMS813160 are also being evaluated in combination with nivolumab, with or without paclitaxel, in clinical trials for colorectal and prostate cancer (NCT03184870) and for non‐small cell lung cancer (NSCLC) and HCC (NCT04123379), aiming to counteract myeloid‐driven immunosuppression [185]. These strategies aim to precisely disrupt tumor‐induced splenic EMH while preserving the spleen's normal physiological functions, offering new directions for combination immunotherapy.

### 5.4 Nanovaccines for Spleen Targeting

Nanovaccines represent a versatile platform for splenic targeting, and their efficacy largely depends on key physicochemical properties such as particle size and surface charge. A size range of 100–200 nm allows them to optimally evade clearance by liver Kupffer cells and RPMs, facilitating efficient accumulation in the splenic MZ and B/T cell areas [186, 187, 188]. Surface charge is equally critical, with negatively charged nanoparticles more likely to accumulate specifically in the spleen [189]. The primary advantages of this platform include excellent biocompatibility and safety profiles compared to viral vectors, the co‐encapsulation of antigen‐encoding mRNA and immunostimulatory adjuvants within a single nanocarrier, and the ability to induce robust, long‐lasting humoral and cellular immune responses. These characteristics make nanovaccines a highly promising strategy for cancer immunotherapy.

Based on these principles, numerous studies have focused on the development of spleen‑targeting lipid nanoparticle (LNP) mRNA vaccines for cancer immunotherapy. For instance, one strategy employed an inflammation‑reduced, spleen‑selective mRNA‑LNP vaccine that enhances splenic mRNA translation and antigen‑specific cellular immunity by dampening the TLR4/MyD88/NF‑κB pathway, leading to strong antitumor efficacy in melanoma models [82]. In a complementary approach, researchers designed spleen‑selective LNPs doped with long‑chain saturated fatty acids to achieve preferential mRNA expression in lymphoid tissues, which improved T‑cell immune responses and showed effective therapeutic and prophylactic activity in mouse lymphoma models [190]. Pan et al. developed stearic acid‐doped anionic LNPs (sLNPs) capable of co‐delivering ovalbumin (OVA)‐encoding mRNA and the Toll‐like receptor 4 (TLR4) agonist MPLA. In a prophylactic mouse model, a single intravenous administration of sLNPs‐OVA/MPLA induced a potent and durable antitumor cellular immune response and demonstrated significant tumor suppression [191]. Another study presents a method for co‐delivering HPV‐16 antigen E7 peptide or E7‐encoding mRNA, and a cyclic GMP‐AMP synthase (cGAS) agonist via LNPs. This strategy enhances anti‐tumor immune responses, reduces immunosuppression, and improves therapeutic efficacy when combined with ICB [192]. Similarly, RNA‐lipoplex (RNA‐LPX) complexes engineered with optimized net charge are efficiently taken up by APCs in the spleen, inducing robust T cell responses in CT26 or B16‐OVA tumor models. A phase I dose‐escalation trial with RNA‐LPX vaccines encoding four tumor antigens (NY‐ESO‐1, MAGE‐A3, tyrosinase, and TPTE) for intravenous administration is currently recruiting patients with advanced malignant melanoma (NCT02410733) [193].

Despite these promising advances, the translation of spleen‐targeting nanovaccines from preclinical models to clinical practice faces several challenges. Batch‐to‐batch variability in nanoparticle formulation complicates large‐scale manufacturing under GMP standards and threatens product consistency, particularly for personalized vaccines. In addition, transfection efficiency in human APCs remains lower than that of viral vectors due to inefficient endosomal escape and lysosomal degradation of nucleic acids. Off‐target accumulation in the liver further reduces splenic bioavailability and may trigger unintended inflammation [194, 195]. To overcome these limitations, ongoing research is therefore focused on developing next‐generation biomaterials with enhanced stability and targeting specificity to fully realize the therapeutic potential of spleen‐targeted nanovaccines [196].

### 5.5 Viral Vectors for Spleen Targeting

Viral vectors represent an effective strategy for splenic‐targeted delivery, primarily due to their high transduction efficiency and inherent immunostimulatory properties, which can be harnessed to enhance anti‐tumor immunity. Modified viral vectors, such as recombinant modified vaccinia virus Ankara (e.g., rMVA‐human IL‐7‐Fc) and human adenovirus serotype, have demonstrated the ability to specifically transduce splenic cells. This leads to a significant expansion of B cells, T cells, NK cells, and myeloid populations in the spleen, underscoring its potent immunostimulatory capacity and therapeutic potential in cancer immunotherapy [165, 166].

The molecular mechanisms of viral vector–mediated spleen targeting involve a combination of intrinsic biophysical properties and engineered modifications. Following intravenous administration, certain AAV serotypes such as AAV9, AAVrh.8, and AAVrh.32.33 are physically retained in the splenic RP through mechanical filtration, facilitated by their small size (∼20–25 nm) and surface characteristics [197]. Engineering of the AAV capsid via directed evolution or rational design enables specific binding to receptors on splenic cells, including DC‐SIGN, CLEC9A, and CD169/Siglec‐1 [198, 199]. Further targeting precision is achieved using cell‐specific promoters (e.g., CD11c for DCs, CD68 for macrophages) or ligand modifications such as mannosylation to engage C‐type lectin receptors, enabling selective transduction of APCs [200]. Lentiviral vectors naturally target the spleen due to their uptake by the reticuloendothelial system. With a size of approximately 80–100 nm, they are efficiently internalized by DCs, macrophages, and B cells [201]. Their tropism can be enhanced by pseudotyping (e.g., VSV‐G) or envelope engineering with single‐chain antibodies or DC‐SIGN ligands for improved delivery to specific subsets such as CD11c^+^ DCs [202, 203].

Currently, lentiviral, adenoviral, and adeno‐associated virus (AAV) vectors are widely explored, each with distinct profiles. For instance, adenoviral vectors induce strong transgene expression and robust T‐cell responses, while some AAV serotypes and engineered lentiviral vectors offer improved preference for splenic APCs [204, 205]. However, the clinical use of viral vectors is limited by their immunogenicity, pre‐existing immunity in patients, and complex manufacturing processes [206, 207, 208]. These challenges can lead to rapid clearance, reduced efficacy, and safety concerns. Consequently, current research focuses on developing next‐generation vectors with lower immunogenicity and enhanced spleen‐targeting capability, as well as exploring non‐viral alternatives [209, 210].

### 5.6 Exploiting RBC for Spleen Targeting

Harnessing the natural splenic homing properties of RBCs represents an innovative biomimetic strategy for achieving efficient drug or vaccine delivery to the spleen. As the primary organ responsible for clearing aged or damaged RBCs, the spleen provides an ideal targeting foundation for RBC‐based delivery systems [211, 212].

To leverage the spleen's natural clearance of RBCs, researchers have developed RBC membrane‐mimetic nanoparticles. By fusing RBC membranes with tumor cell membranes or mannosylated liposomes, these nanovaccines selectively accumulate in the spleen, activate DCs and T cells, and elicit potent anti‐tumor immunity [213, 214]. A more sophisticated approach is erythrocyte‑driven immune targeting (EDIT), where antigen‑loaded nanoparticles are attached to RBC surfaces for precise delivery to splenic APCs. Optimizing the nanoparticle‑to‑RBC ratio maximizes splenic accumulation and minimizes off‑target retention. Studies have shown that the EDIT strategy induces stronger neoantigen‐specific T cell responses and humoral immunity, and when combined with PD‐1 blockade in models such as HCC, can even lead to complete tumor regression [167, 168].

Beyond antigen and adjuvant delivery, RBC‐based platforms are being engineered to directly modulate critical immune checkpoints within the spleen. Nie et al. reported a novel RBC‑anti‑PD‑1 antibody conjugate (aPD‑1‑Ery) that targets the splenic immune microenvironment to systemically reshape anti‐tumor immunity. This strategy demonstrated favorable safety and significant antitumor activity in both preclinical models and a first‑in‑human phase I trial involving patients with advanced, ICB‑resistant solid tumors, offering a promising approach to overcome ICB resistance [215]. In addition, various RBC‐based artificial APCs (aAPCs) have been developed to enhance immune activation. For example, DNA‐directed assembly was used to attach pMHC complexes and CD28 antibodies to RBCs for effective T cell activation [216]. Sun et al. employed a biotin‐avidin bridging system to co‐modify RBCs with pMHC complexes, CD28 antibody, and PEG‐linked IL‐2, boosting proliferation and cytokine secretion of antigen‐specific CD8^+^ T cells [217]. Zhang et al. achieved in vivo T cell expansion and tumor control by co‐displaying pMHC complexes, 4‐1BBL, and IL‑12 [218]. Another study used click chemistry to equip RBCs with 4‐1BBL and IL‐15, enabling simultaneous activation of T cells and NK cells [219]. Recently, mgSrtA‑catalyzed conjugation of MHC‑I molecules to RBCs showed potent anti‑tumor efficacy, offering a novel RBC‑based vaccine approach [169].

These RBC‐based splenic targeting strategies skillfully leverage the body's natural physiological processes, opening new avenues for enhancing the precision and efficacy of cancer immunotherapy. The key advantages of this approach include superior biocompatibility, extended circulatory half‐life, and intrinsic spleen‐targeting capability through the natural RBC clearance pathway, enabling efficient antigen presentation and immune activation [21, 220]. However, limitations remain, such as constrained drug‐loading capacity, potential instability of encapsulated therapeutic agents, and challenges in scalable manufacturing and quality control [216, 219]. Current research is actively addressing these hurdles through novel engineering techniques to fully realize the potential of this promising platform.

### 5.7 Engineering Neutrophils for Spleen Targeting

**FIGURE 4:**
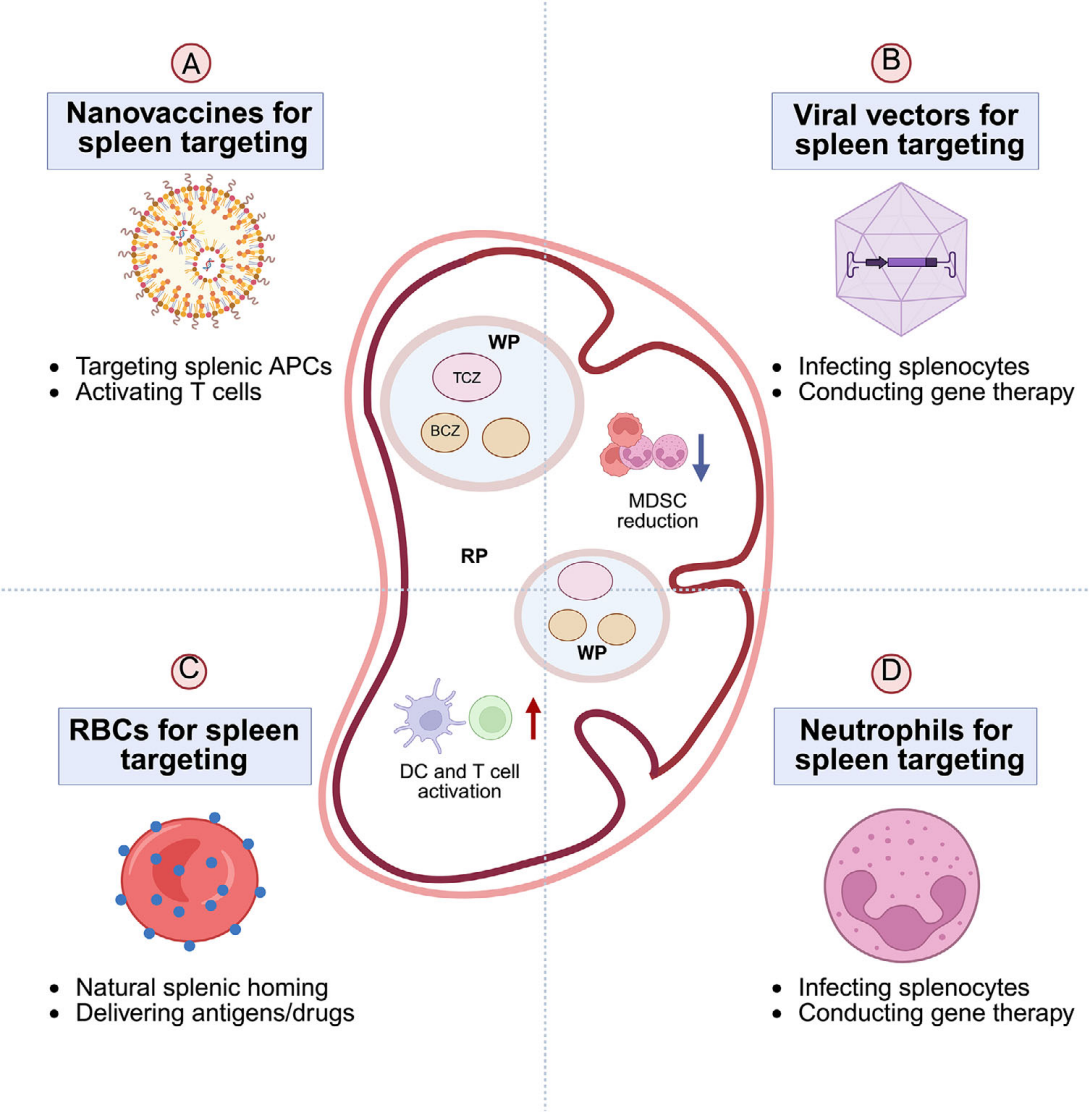
Spleen‐targeted cancer therapy strategies. (A) Nanovaccines (e.g., LNPs) deliver mRNA antigens and TLR4 agonists to splenic APCs, activating T cells. (B) Viral vectors (e.g., rMVA‐IL‐7‐Fc, Ad49) target splenic APCs to induce T‐cell responses. (C) RBCs act as “shuttles” to deliver drugs to the splenic RP, targeting APCs and macrophages. (D) Neutrophils loaded with therapeutics home to the spleen via chemotaxis. Created in https://BioRender.com.

Neutrophils represent a promising delivery vehicle for splenic targeting due to their intrinsic chemotactic properties. Studies have shown that intravenous injection of neutrophils loaded with nanoparticles (NPs@NEs) can alter the in vivo distribution of nanoparticles, leading to their specific accumulation in the spleen [170]. This process is mediated by the CCL9/CCR1 chemokine axis. Splenic stromal cells secrete CCL9, which binds to CCR1 on neutrophils and directs their migration to the spleen [52]. The neutrophil‐based delivery platform offers distinct advantages, including high targeting precision through natural chemotaxis and enhanced splenic penetration [221]. However, its clinical translation faces challenges such as the short lifespan, difficulties in maintaining cell viability during manipulation, and potential risks of triggering inflammatory responses [222, 223]. Current research aims to extend neutrophil longevity and optimize loading techniques to enhance therapeutic utility [224, 225]. Together, the strategies outlined above provide approaches for achieving precise spleen‐specific drug delivery, as summarized in Figure 4.

## 6 Challenges and Perspectives of Spleen‐Targeted Therapies

While spleen‐targeted strategies hold great therapeutic potential, their translation from basic research to clinical application faces multiple challenges, including dominant hepatic sequestration, RPM clearance, limited WP access, low intracellular delivery efficiency, and pronounced species differences. One key challenge is biological distribution. Most nanovaccines are quickly cleared by liver Kupffer cells or filtered by the kidneys after intravenous administration [153, 154]. Though adjusting particle size and surface charge can improve targeting, accumulation in the spleen is only modestly higher than in the liver, which means most of the dose is still sequestered in hepatic tissue [213, 226]. This limited efficiency also increases the risk of hepatotoxicity [227, 228, 229].

Another barrier is within the spleen itself. As soon as nanovaccines enter the blood, they bind plasma proteins and form a protein corona, which alters their surface properties and promotes uptake by macrophages [230]. RPMs in the spleen remove a significant portion of particles, making it difficult for vaccines to reach TCZs and BCZs in the WP [231]. Even when appropriately sized particles reach lymphoid areas, intracellular delivery remains inefficient. For instance, less than 2% of mRNA escapes the endosome in LNP systems, limiting antigen expression and immune activation [232].

Clinical application also faces biosafety and species‐specific issues. Delivering higher doses or prolonging treatment can trigger chronic inflammation and vascular damage, which may even support tumor metastasis [233, 234]. There are also anatomical differences between human and mouse spleens. The human MZ is smaller and contains fewer B cells, which may restrict the direct translation of preclinical findings [235, 236].

Manufacturing challenges add further complexity. Surface modifications may increase targeting but also raise costs and formulation difficulty. Glybera, the first gene therapy, was discontinued due to high production expenses and poor commercial viability [237, 238]. Achieving reliable quality control, scalable production, and consistent batches remains essential for industrial translation. Addressing these challenges will be crucial for moving spleen‐targeted therapies forward in treating cancer, autoimmune, and other immune‐mediated diseases.

## 7 Unresolved Questions, Biomarkers, and Pathways to Clinical Translation

Despite increasing insights into the role of tumor‐induced splenic remodeling in systemic immunosuppression, and the promising potential of spleen‐targeted therapeutic strategies, translating these scientific advances into effective clinical therapies remains challenging. This section outlines key unresolved scientific questions, discusses potential biomarkers for monitoring splenic reprogramming, and analyzes major barriers to clinical translation.

### 7.1 Unresolved Scientific Questions

The regulatory network governing splenic remodeling and its role in cancer immunity remains incompletely defined. TDFs reprogram splenic homeostasis through systemic inflammatory signals such as GM‐CSF, G‐CSF, and IL‐6, though potential upstream or spleen‐specific initiators are not fully identified [17, 239, 240]. Stromal and immune cell subsets interact within the splenic niche to drive pathological EMH and MDSC expansion, but their precise crosstalk is unclear [41, 52]. Organ‐specific communication axes between the spleen, bone marrow, and TME may coordinate systemic immunosuppression, yet their existence and mechanisms require validation. Additionally, anatomical and immunological differences between mouse and human spleens pose significant barriers to clinical translation.

### 7.2 Potential Biomarkers for Monitoring Splenic Remodeling

The development of non‐invasive biomarkers is crucial for patient stratification and treatment monitoring. Circulating biomarkers may include the frequency and absolute count of M‐MDSCs and PMN‐MDSCs in peripheral blood, although their specificity and assay standardization require improvement [241, 242]. The detection of signature proteins, circulating cell‐free DNA methylation patterns, or RNA profiles specifically released by splenic immunosuppressive cells may offer more precise insights into systemic immune modulation. In imaging, novel positron emission tomography (PET) tracers targeting spleen‐enriched immunosuppressive populations enable non‐invasive assessment of splenic immune status, especially when combined with magnetic resonance imaging (MRI) or computed tomography (CT) for structural evaluation of spleen volume and texture. For patients undergoing splenectomy, ex vivo analysis of splenic tissue by immunohistochemistry, multiplex immunofluorescence, or spatial transcriptomics can provide a “gold standard” for biomarker validation.

### 7.3 Key Challenges in Clinical Translation

Clinical translation of spleen‐targeted therapies faces multiple hurdles. Technically, despite ongoing optimization of nanovaccines and viral vectors, rapid hepatic clearance and uptake by RPMs in the spleen remain major barriers, resulting in low delivery efficiency and insufficient intracellular release [153, 154]. Safety concerns include immune homeostasis disruption and potential inflammatory or autoimmune reactions, while preserving the spleen's physiological functions remains critical. Patient identification is challenging due to variable splenic remodeling, necessitating reliable biomarkers. Optimal combination strategies with existing immunotherapies require further exploration. Translational relevance is hampered by anatomical differences between murine and human spleens, underscoring the need for humanized models. Finally, manufacturing complexity, cost, and batch consistency pose significant barriers to scalable production.

## 8 Conclusion

During tumor progression, the spleen acts as a critical reservoir for tumor‐promoting immunosuppressive cells. Through EMH, the spleen becomes a major production site for MDSCs. These cells are not only generated and stored in the spleen but are also continuously supplied to the TME. Within the spleen, MDSCs locally suppress anti‐tumor T‐cell activity by expressing high levels of inhibitory molecules like PD‐L1. Once trafficked to tumors, they exert powerful systemic immunosuppression by secreting factors (e.g., ARG1, iNOS, TGF‐β) that inhibit effector T and NK cells, promote Treg expansion, and recruit other immunosuppressive populations like TAMs. The coordinated immunosuppressive environment in the spleen and TME interferes with T cell responses, impairs the effect of ICB, and enables tumor immune escape.

Therefore, directly targeting the spleen to reprogram its immunosuppressive function represents a rational and promising therapeutic strategy. By intervening at this systemic source, it is possible to disrupt the sustained supply of MDSCs, thereby alleviating both local and systemic immunosuppression and restoring the efficacy of ICB. This approach expands the therapeutic focus beyond the TME by targeting the systemic origin of immunosuppressive cells. By disrupting their generation in the spleen, it addresses a key mechanism of immunotherapy resistance and supports the rationale for splenic targeting in combination strategies. Encouragingly, emerging technologies such as spleen‐targeted nanovaccines and cell‐mimetic delivery systems hold great promise for enabling precise modulation of the splenic immune environment. However, the clinical translation of these cutting‐edge approaches still faces significant challenges. Increased complexity in manufacturing processes may compromise treatment timeliness, and key technical bottlenecks in delivery efficiency, biosafety, and species differences urgently need to be overcome. Therefore, future research must carefully evaluate the balance between therapeutic efficacy and clinical applicability.

A deeper understanding of the dynamic regulatory mechanisms in the spleen is essential. Cross‐disciplinary collaboration among immunology, nanotechnology, and translational medicine will be critical to overcoming current barriers to clinical translation. As knowledge of the splenic immune network grows and innovative delivery platforms mature, opportunities to expand therapeutic windows for cancer patients are likely to emerge. Rationally designed spleen‐targeted strategies may thus play an increasingly important role in future immunotherapies.

## Conflicts of Interest

X.G. is a founder of Westlake Therapeutics Co., Ltd., and a member of its scientific advisory board. The remaining authors declare no competing interests.
